# Efflux Pump Activity and Mutations Driving Multidrug Resistance in *Acinetobacter baumannii* at a Tertiary Hospital in Pretoria, South Africa

**DOI:** 10.1155/2021/9923816

**Published:** 2021-10-07

**Authors:** Noel-David Nogbou, Granny M. Nkawane, Khanyisa Ntshane, Charles K. Wairuri, Dikwata T. Phofa, Kagiso K. Mokgokong, Mbudzeni Ramashia, Maphoshane Nchabeleng, Lawrence C. Obi, Andrew M. Musyoki

**Affiliations:** ^1^Department of Microbiological Pathology, School of Medicine, Sefako Makgatho Health Sciences University, Pretoria, South Africa; ^2^Microbiology Unit, National Health Laboratory Services, Dr George Mukhari Academic Hospital, Pretoria, South Africa; ^3^School of Science and Technology, Sefako Makgatho Health Sciences University, Pretoria, South Africa

## Abstract

*Acinetobacter baumannii* (*A*. *baumannii*) has developed several resistance mechanisms. The bacteria have been reported as origin of multiple outbreaks. This study aims to investigate the use of efflux pumps and quinolone resistance-associated genotypic mutations as mechanisms of resistance in *A*. *baumannii* isolates at a tertiary hospital. A total number of 103 *A*. *baumannii* isolates were investigated after identification and antimicrobial susceptibility testing by VITEK2 followed by PCR amplification of *bla*_*OXA-51*_. Conventional PCR amplification of the AdeABC efflux pump (*adeB*, *adeS*, and *adeR*) and quinolone (*parC* and *gyrA*) resistance genes were performed, followed by quantitative real-time PCR of AdeABC efflux pump genes. Phenotypic evaluation of efflux pump expression was performed by determining the difference between the MIC of tigecycline before and after exposure to an efflux pump inhibitor. The Sanger sequencing method was used to sequence the *parC* and *gyrA* amplicons. A phylogenetic tree was drawn using MEGA 4.0 to evaluate evolutionary relatedness of the strains. All the collected isolates were *bla*_*OXA-51*_-positive. High resistance to almost all the tested antibiotics was observed. Efflux pump was found in 75% of isolates as a mechanism of resistance. The study detected *parC* gene mutation in 60% and *gyrA* gene mutation in 85%, while 37% of isolates had mutations on both genes. A minimal evolutionary distance between the isolates was reported. The use of the AdeABC efflux pump system as an active mechanism of resistance combined with point mutation mainly in *gyrA* was shown to contribute to broaden the resistance spectrum of *A*. *baumannii* isolates.

## 1. Background

*Acinetobacter baumannii* (*A*. *baumannii*) is one of the nosocomial pathogens that exhibits high level of resistance to antibiotics [1, 2]. This Gram-negative coccobacillus bacterium is responsible for infections predominantly in the intensive care unit [3, 4]. The bacterium causes pneumonia, bacteraemia, septicaemia [5], meningitis [6], and urinary tract infections [7] often seen in patients undergoing invasive procedures such as urinary catheter, tracheal intubation, and those with underlying conditions [2]. *A*. *baumannii* has demonstrated several antimicrobial resistance mechanisms [8–10] against antibiotics such as aminoglycosides, carbapenems, fluoroquinolones, cephalosporins, tetracyclines, sulbactams, rifampicins [9], as well as colistin [11–13] and tigecycline [8, 14], the so called last resort drugs used to treat its infections [8, 11–14]. Moreover, literature has reported resistance to recently developed antibiotics such as eravacycline [15] and cefiderocol [16]. The bacterium has acquired several resistance mechanisms through mobile genetic elements [17, 18] and has also shown to have natural resistance against some antibiotics including aminopenicillins, first and second generation cephalosporins, aztreonam, ertapenem, fosfomycin, chloramphenicol, and trimethoprim [19] leading to emergence of multidrug and pandrug-resistant strains [8, 9]. The main mechanisms expressed by *A*. *baumannii* to escape antibiotics attack are production of antibiotic-hydrolysing enzymes, poor membrane penetration, permeability defects, alteration of target sites or cellular functions, and active efflux pumps [20].

Efflux pumps are the main mediators of resistance mechanisms against many antibiotic classes [21]. Through this mechanism, the bacteria avoid accumulation of drugs at the targeted site within it, leading to decreased susceptibility to antibiotics [17, 21]. Three resistance-nodulation-cells division encoded in *A*. *baumannii* genome is reported to contribute to antibiotic resistance in *A*. *baumannii* clinical isolates [22]. The overexpression of efflux pumps in *A*. *baumannii* has been associated with an increased resistance to antibiotics such as tigecycline [14, 23], carbapenems [24], minocycline, gentamycin, doxycycline, and tetracycline [25]. Several researchers support that among the resistance-nodulation-cell division superfamily, the adverse effect of ATP-binding cassette (AdeABC) is the most associated with resistance in *A*. *baumannii* [26, 27]. The system generally consists of three parts: multidrug transporter *adeB* that captures antibiotics in the inner membrane of phospholipids bilayer or cytoplasm, membrane fusion protein *adeA* that acts as membrane fusion protein, and finally, the outer membrane protein *adeC* that is a membrane channel protein, used by *adeB* to transport out the substrates [26]. This whole mechanism is regulated by *adeR* and *adeS*, a two-component system [26, 27]. The working mechanism of this system suggests that *adeB* and the regulatory genes *adeR* and *adeS* are the main role players within the AdeABC efflux pump system [28]. This mechanism of resistance reduces the susceptibility of *A*. *baumannii* to multiple class of antibiotics [23, 28–30] and has been reported to be associated with resistance to newly developed drugs [30].

Alteration of target site or cellular function due to mutations has enabled emergence of resistance to antibiotics within the *A*. *baumannii* species [15, 31]. This mechanism of resistance is used by *A*. *baumannii* to resist fluoroquinolones [32], colistin [33], novel synthetic beta-lactamase ceftazidime–avibactam [34], and several newly developed antibiotics [35]. Even though literature reports on the plasmid mediated quinolone resistance genes *qnrA*, *qnrB*, and *qnrS* as one of the resistance mechanisms to reduce susceptibility to quinolones [36, 37], *A*. *baumannii* are mainly resistant to quinolones through chromosomal gene mutation in *parC* and *gyrA* [38, 39] and other molecular mechanisms [37]. Literature supports that a single mutation in *gyrA* inducing an amino acid change from serine to leucine in position 83 (serine 83) reduces the susceptibility of A. *baumannii* clinical isolates to fluoroquinolones [40]. Two mutations in *parC* in position 80 (serine 80) and 84 (Glu-84) inducing change from serine to isoleucine and glutamic acid into valine, respectively, lead to resistance to fluoroquinolones [40].

The combined effect of active use of efflux pumps and alteration of target site due to mutations contribute to broaden the resistance spectrum of *A*. *baumannii* strains and pose serious therapeutic challenges to clinicians in establishment of effective treatment regime. Multiple outbreaks of *A*. *baumannii* infections have been observed globally with an increased resistance to antimicrobial drugs [41, 42]. In this study, we focused on the use of AdeABC efflux pumps, *parC* and *gyrA* mutations, contributing to reduce *A*. *baumannii* clinical isolates susceptibility to antibiotics at an academic hospital in Pretoria. The aim was to investigate the use of the efflux pump and quinolone resistance-associated genes in the rise of multidrug resistant *A*. *baumannii* strains.

## 2. Materials and Methods

### 2.1. Study Design, Settings, and Samples Collection

Isolates for this study were collected between February 2018 and February 2020 at Dr. George Mukhari Tertiary Laboratory (DGMTL), a unit of the National Health Laboratory Services (NHLS) of South Africa. DGMTL is a level 3 clinical laboratory where routine laboratory diagnostics for patients presenting at Dr. George Mukhari Academic Hospital (DGMAH), and 3 district hospitals and surrounding clinics are performed. DGMTL is coupled with the Department of Microbiological Pathology of Sefako Makgatho Health Sciences University (SMU). Ethical approval to conduct this research was granted by Sefako Makgatho Health Sciences University Research Ethics Committee (SMUREC). A total number of 103 *A*. *baumannii* isolates were collected from DGMTL and stored at −70^o^C until use.

### 2.2. Isolate's Identification and Antimicrobial Susceptibility Profiles

Collected isolates were identified using a phenotypic and genotypic method, VITEK2 automated system (bioMerieux, France) and polymerase chain reaction (PCR) amplification of *bla*_*OXA-51*_ gene.

Antimicrobial susceptibility testing was performed using the VITEK2 automated system (bioMerieux, France). Piperacillin + tazobactam (ptz), ceftazidime (caz), cefepime (fep), trimethoprim/sulfamethoxazole (sxt), gentamycin (cn10), ciprofloxacin (cip), cefotaxime (ctx), imipenem (imp), meropenem (mem), and tigecycline (tig) were tested.

### 2.3. Molecular Investigation of Resistance Mechanisms

#### 2.3.1. Recovery of Isolates and Nucleic Acid Extraction

Recovery of a fresh pure colony on Muller–Hinton (MH) agar (Diagnostic Media Products, DMP, South Africa) was conducted from microbanks (Microbanks^™^, ProLab Diagnostics Inc., Canada) under aseptic environment. A single pure colony was used for DNA and RNA extraction. DNA was extracted using the boiling method [43]; briefly, a loopful of fresh pure colony was suspended in 1000 *µ*L of saline (SABAX Pour Saline 0.9%, Adcock Ingram Critical Care (Pty) Ltd., South Africa) in an Eppendorf tube (Eppendorf AG, Hamburg, Germany) followed by incubation (Memmert Incubator IN30, Germany) for 20 min at 37°C ± 2. The suspension was then removed and well shaken using a vortex (Vortex, Heidolph, Reax top, Germany) at full speed for 20 s. Thereafter, the suspension was centrifuged (Mikro 20, Werk Nr. Bajahr E Kin, Hettich Zentrifugen, Germany) for 5 min at 130 × 100 rotation per minute (RPM). The supernatant was removed and resuspended in 200 *µ*L of PCR grade water (BioConcept Ltd., Switzerland) in an Eppendorf tube (Eppendorf AG, Hamburg, Germany). The suspension was then put on a thermomixer (Eppendorf, Thermomixer Compact, MERCK, Merck Chemical (Pty) Ltd., South Africa) at 90°C for 10 mn at 700 RPM. Thereafter, it was put on a cell disruptor (Disruptor Genie^®^, Scientific Industries SI^™^, USA) for 10 min. Finally, the suspension was centrifuged (Mikro 20, Werk Nr. Bajahr E Kin, Hettich Zentrifugen, Germany) for 10 min at 130 × 100 RPM. The supernatant containing the DNA was transferred and preserved in the sterile Eppendorf tube (Eppendorf AG, Hamburg, Germany) and stored at −20°C.

RNA extraction was conducted using a commercial RNA isolation kit (ISOLATE II RNA Mini Kit, MagMAX^™^ Viral/Pathogen, bioline, London, United Kingdom) following the manufacturer's instructions [44]. Extracted RNA was quantified using a spectrophotometer (NanoDrop Lite Spectrophotometer, NanoDrop Products, Thermo Scientific, USA) and normalized to 1 *μ*g/*μ*L. The obtained RNA was stored at −80°C. A complementary DNA (cDNA) synthesis was performed using a cDNA synthesis kit (SensiFAST cDNA Synthesis Mix Reaction Guidelines, Bioline, a Meridian Life Science^®^ Company, United Kingdom) following the manufacturer's instructions. cDNA products were stored at −20°C, until further use.

#### 2.3.2. Conventional PCR Amplification of AdeABC Efflux Pump (*adeB*, *adeS*, and *adeR*) and Quinolone (*parC* and *gyrA*) Resistance Genes

Gene amplification by conventional PCR was performed using 2X MyTaq HS Red Mix (MyTaq HS Red Mix, Meridian Bioscience, bioline, United Kingdom) to detect genes of interest in *A*. *baumannii*. The Bioline protocol [45] was followed to prepare multiplex PCR (M-PCR) assays using primers with similar melting temperatures and monoplex PCR (m-PCR) for primers of different annealing temperatures. To avoid mistakes during detection of amplicon by gel electrophoresis, the AdeABC efflux pump system associated genes *adeB* and *adeS* which differ by only 3 nucleotides in base pairs were ran separately. M-PCR was run for genes *adeS* and *adeR* and a m-PCR for gene *adeB*. PCR was performed in a reaction mixture of a total volume of 25 *μ*L, composed as follows: 12.5 *μ*L of 2X MyTaq HS Red Mix (MyTaq HS Red Mix, Meridian Bioscience, Bioline, United Kingdom), 0.5 *μ*L of each primer, and 6.5 *μ*L PCR grade water (BioConcept Ltd., Switzerland) were added to make up to 20 *μ*L, and 5 *μ*L of DNA template was added to constitute a 25 *μ*L reaction mix. Negative and positive controls were run with every PCR on a thermocycler (GeneAmp^®^ PCR System 2700, Applied Biosystems, Singapore). The thermocycling conditions for conventional PCR and primers used for detection of drug resistance-associated genes are detailed in Supplementary 2.

#### 2.3.3. PCR Amplicon Detection

The detection of PCR amplicons was conducted in a 1% agarose gel stained with ethidium bromide.

#### 2.3.4. Quantitative Real-Time PCR (qRT-PCR) Amplification of AdeABC Efflux Pump (*adeB*, *adeS*, and *adeR*)

The qRT-PCR was conducted on cDNA for the detection of the presence of mRNA, using the SYBR No-ROX kit (SensiFAST^™^ SYBR^®^ NO-ROX Kit, Meridian Bioscience, Bioline, United Kingdom) on Sacace Real-Time PCR Instrument (SaCycler-96, Real-Time PCR System, Sacace Biotechnologies Srl, Scalabrini, Italy). The qRT-PCR reaction mixture was prepared in a volume of 20 *μ*L comprising of 4 *μ*L of cDNA, 10 *μ*L SYBR, 0.8 *μ*L of each primer, and 4.4 *μ*L of PCR grade water (BioConcept Ltd., Switzerland). The thermocycling conditions and primers used are detailed in Supplementary 1 and 2, respectively.

### 2.4. Phenotypic Evaluation of AdeABC Efflux Pump *adeB*, *adeS*, and *adeR* Gene Expression

A functional AdeABC efflux system was assessed by evaluating the difference between the minimal inhibitory concentrations (MICs) for tigecycline (TGC) using the gradient diffusion method (tigecycline, MIC Test Strip, Liofilchem^®^ Srl, Roseto d'Abruzzi, Italy) before and after exposure to an efflux pump inhibitor (EPI) carbonyl cyanide 3-chlorophenylhydrazone (CCCP) (Sigma-Aldrich, Dorset, United Kingdom) [46]. Briefly, CCCP was added to one of two MH agar plates at the final concentration of 100 *µ*g/mL. From a fresh overnight culture of *A*. *baumannii*, a 0.5 Mac Farhland turbid sample was made (Densichek, BioMerieux DensiCHEK Plus, USA). A swab was then used to spread *A*. *baumannii* on each agar plate, followed by placement of a 0.016–256 mg/L TGC E-strip test in the middle of each agar plate followed by an overnight incubation at 37°C. *E*. *coli* strain ATCC 85218 was used as a control.

### 2.5. Sequencing of Quinolone Resistance-Associated Genes (*parC* and *gyrA*)

The Sanger sequencing method (ABI3500XL; Applied Biosymptoms, United States) at Inqaba Biotec (Pretoria) was used to sequence the *parC* and *gyrA* amplicons. Sequences were edited using ChromasPro software (version 2.0) and then aligned together with wild-type sequences from GenBank using BioEdit (http://www.mbio.ncsu.edu/BioEdit/BioEdit.html). Serine 83 of *gyrA* gene and serine 80 and serine 84 of *parC* gene were investigated for mutation. A phylogenetic tree was drawn using molecular evolutionary genetic analysis (MEGA) 4.0 [47] to evaluate evolutionary relatedness of the study strains.

### 2.6. Statistical Analysis

Data were captured on Microsoft Professional Excel 2016 and data analysis conducted on IBM SPSS Statistics 26 (IBM® SPSS® Statistics version 26.0, 2019). A *p* value of ≤0.05 was considered statistically significant.

## 3. Results

### 3.1. Isolate Identification and Antimicrobial Susceptibility Profile

**Figure 1:**
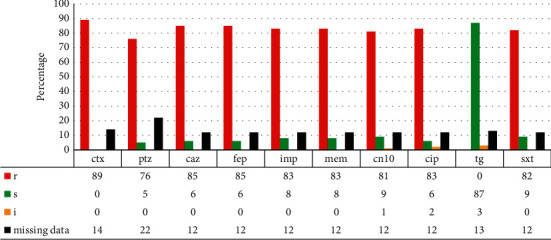
Antimicrobial susceptibility testing.

All *A*. *baumannii* isolates identified by VITEK2 were PCR-positive for *bla*_*OXA-51*_. The antibiotic susceptibility of the collected isolates was tested against 10 commercially available antibiotics. The study isolates were 89% resistant to cefotaxime and 85% resistant to both ceftazidime and cefepime (Figure 1). The isolates were 76% resistant to piperacillin + tazobactam, a combination of penicillin and beta-lactamase inhibitor (Figure 1). Eighty-three percent (83%) of the isolates were resistant to imipenem and meropenem (Figure 1), while resistance to gentamycin, trimethoprim-sulfamethoxazole, and ciprofloxacin was 81%, 82%, and 83%, respectively (Figure 1). Antibiotic susceptibility testing for tigecycline showed that 87% of isolates were susceptible, while 3% demonstrated intermediate susceptibility (Figure 1).

### 3.2. Molecular and Phenotypic Evaluation of the Active AdeABC Efflux Pump System as the Resistance Mechanism

The evaluation of an active AdeABC efflux pump system as a mechanism of resistance was conducted using a combination of genetic and phenotypic tests targeting *adeB*, *adeR*, and *adeS* genes. A positive PCR and qRT-PCR of targeted genes suggest that the genes of interest are present and that the related proteins are actively produced. The phenotypic evaluation confirms the actual use of the efflux pump system as the resistance mechanism at the phenotypic level.

The positive PCR amplification of targeted genes was 100% for *adeB* and 99% for both *adeR* and *adeS* genes (Table 1; Supplementary 3). The qRT-PCR was 100% positive for *adeB* and 99% and 98.1% for *adeR* and *adeS*, respectively. A total of 100 isolates (97%) were positive for PCR and qRT-PCR targeting *adeB*, *adeR*, and *adeS* genes. Among the 100 isolates, 75% of these isolates phenotypically demonstrated the active use of efflux pumps as a drug resistance mechanism (Table 1; Supplementary 3) and 25% did not show any phenotypic level of efflux pump involvement in resistance to tigecycline. Of the 25% that did not demonstrate phenotypic expression of efflux pump use, 88% (22 isolates) had the complete required set of genes for an active efflux pump (Table 1; Supplementary 3). There was a statistically significant association between positive conventional PCR and qRT-PCR amplification of structural *adeB* and regulatory *adeR* genes and active phenotypic expression of the efflux pump (*p* value <0.05).

### 3.3. Investigation of Point Mutations in *parC* and *gyrA*

**Figure 2:**
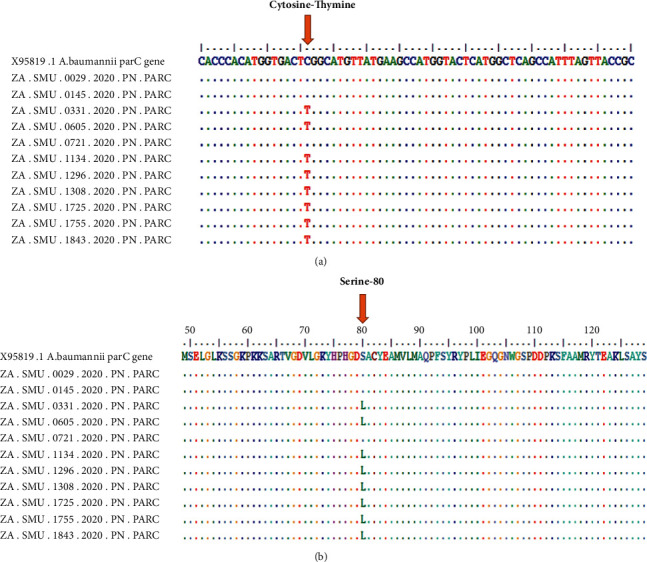
Sequences analysis of *parC* gene.

**Figure 3:**
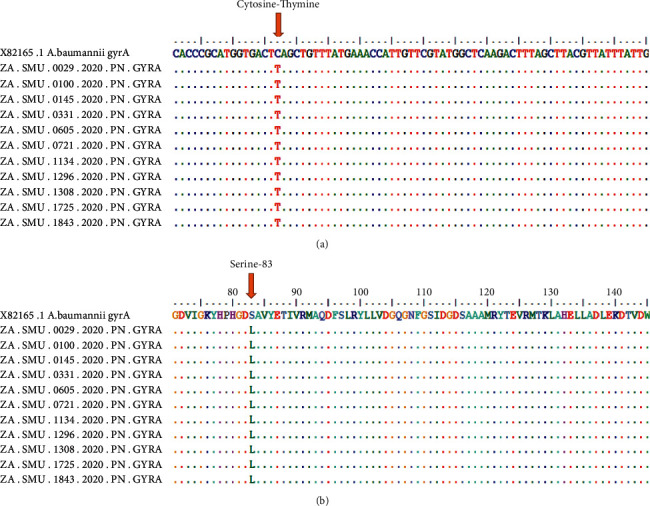
Sequences analysis of *gyrA* gene.

The point mutations in *parC* and *gyrA* genes were investigated following Sanger sequencing of PCR products. All study isolates (100%) were PCR-positive for *gyrA* and 99% positive for *parC* (Table 2; Figure 2; Supplementary 4), while 2 samples failed quality control for *gyrA* Sanger sequencing and could not be sequenced. The sequence analysis of *gyrA* revealed that 89% of the isolates showed a point mutation in serine 83 inducing a change in amino acid from serine to leucine (Table 2; Figure 3; Supplementary 4), while 11% did not have this mutation. All the isolates (100%) were negative for point mutation on serine 84 of *parC* gene; while on serine 80, 39% of isolates had a point mutation inducing change in amino acid from serine to leucine. This point mutation was not observed in 61% of the isolate sequences that were analysed (Table 2; Figure 3; Supplementary 4). The analysis of the two gene sequences revealed that 36% (37 isolates) had both point mutations. Serine 83 for *gyrA* and serine 80 for *parC* and 10 isolates did not have any of the mutations (Table 2; Supplementary 4).

The occurrence of mutations among isolates collected has increased over time. In 2017, *parC* mutation among collected isolates was 5% (1/22), 36% (14/32) in 2018, and 60% (25/42) in 2020 (Table 2; Figure 3; Supplementary 4). *gyrA* mutation in isolates showed a similar trend, 86% (19/22), 95% (37/39), and 85% (34/40) in 2017, 2018, and 2020, respectively (Table 2; Figure 3; Supplementary 4).

## 4. Discussion

*Acinetobacter baumannii* increasing the spectrum of resistance to available antibiotics is of a public health concern [1]. Similar to previous studies, PCR amplification of *bla*_*OXA-51*_ gene was used as a genotypic identification and confirmatory method for *A*. *baumannii* strains previously identified by VITEK2 (bioMerieux, France) [49–51]. Even though it has been reported in literature that some of *A*. *baumannii* strains do not harbour *bla*_*OXA-51*_ [53], the gene has been reported intrinsic to the species by several authors [49–51, 53]. The use of *bla*_*OXA-51*_ has been recommended as a simple and reliable identification method for *A*. *baumannii* strains [49–51].

This study results reveal a high resistance to most of available antibiotics used by clinicians in management of *A*. *baumannii* infections (cefotaxime 89%, ceftazidime 85%, cefepime 85%, piperacillin + tazobactam 76%, imipenem 83%, meropenem 83%, ciprofloxacin 83%, gentamycin 81%, and trimethoprim-sulfamethoxazole 82%) (Figure 1). The findings are similar to several reports made by different researchers within the region [54–56] and globally [58]. In their published report on the district of Oliver Reginald Tambo in the Eastern Cape Province of South Africa, Anane et al. [55] highlighted that *A*. *baumannii* strains showed resistance rates above 80% against the same antibiotics tested in this current study. Similarly, Lowe et al. [57] in Tshwane district in Gauteng province of South Africa reported high prevalence of resistance (69–90%) of *A*. *baumannii* strains to the same antibiotics tested in the current study.

An increasing number of isolates with an intermediate susceptibility to tigecycline (3%) is of concern (Figure 1). A raise of resistance to last resort antibiotics against *A*. *baumannii* suggests that soon there will be no antibiotics with effective antibacterial action against locally circulating strains of *A*. *baumannii*. This report is another call to support cautious prescription and use of antibiotics in accordance with local and international guidelines [59] and stress the need to develop a new antimicrobial alternative against this bacterium.

The evaluation of the AdeABC efflux pump system as a mechanism of resistance used by *A*. *baumannii* strains isolated at DGMTL revealed a high prevalence of *adeB* (100%), *adeR* (99%), and *adeS* (99%). This is in agreement with other studies that reported the AdeABC efflux pump system as the most prevalent and with the highest detection rate in clinical isolates compared to other efflux pump genes [26]. This may be explained by the physiologic role of the efflux pumps which are proteins on the bacterial cell membrane that regulate the movement of substances from the internal to the external cell environment [60]. However, a high prevalence of the AdeABC efflux pump in bacteria clinical isolates may be explained by the involvement of the pump in driving resistance against antibiotics as hypothesized by Ranjbar and his colleagues [61]. A study conducted in China demonstrated that the AdeABC efflux pump is responsible for an increase in multidrug-resistant *A*. *baumannii* strains in their paediatric intensive care unit [42]. Similar findings were reported in Iraq where the AdeABC efflux pump system was 96% prevalent in clinical isolates of *A*. *baumannii* [61]. Mahmoudi et al. [62] also mentioned that AdeABC efflux pump system genes were present in more than 90% of *A*. *baumannii* clinical isolates in their study. The positive statistical association of structural *adeB* and regulatory *adeR* genes may be explained by the molecular structural organisation of the AdeABC operon system regulated by *adeRS*. The *adeR* gene has been identified as a recognition response factor that acts as a transcriptional activator under the influence of *adeS* [26]. However, exploring the interaction between *adeR* and *adeABC*, Chang et al. [63] reported that *adeR* action can be independent of *adeS* influence. Their study revealed that *adeR* gene may be activated as a result of an amino acid substitutions in a repeat motif region between *adeR* and *adeABC* leading to *adeABC* overexpression and increase tolerance to antibiotic action. The complete understanding of the AdeABC efflux pump system working and regulation mechanism is still limited. Even though additional research is needed to explore the regulation of this mechanism of resistance, this report is inclined to support the finding by Chang et al. [63], whereby, despite the presence of *adeS*; some AdeABC efflux pumps system in the study isolates did not demonstrate phenotypic resistance to tigecycline (Table 1; Supplementary 3). This study data revealed that 97% (100/103) of the isolates had the required set of genes (*adeB*, *adeR*, and *adeS*) for expression of an active efflux pump (Table 1; Supplementary 3). Of these isolates, 75% (75/100) demonstrated an active use of the AdeABC efflux pump as a mechanism of resistance, while 25% (25/100) did not demonstrate at phenotypic level expression of the AdeABC efflux pump as a mechanism of resistance. Literature supports that it is not just the presence of the AdeABC efflux pump-associated gene but their overexpression triggered by a mutation that induce resistance to antibiotics [15, 63, 64]. Furthermore, it is documented that *A*. *baumannii* can use other efflux pump-associated genes [14, 65] and superfamily [22] to develop a multidrug-resistance profile. In addition, researchers reported that the AdeABC efflux pump need, in some cases, a synergistic action with other resistance mechanisms to express a level of resistance to particular antibiotic classes [67]. All these may explain the phenotypic result of 22/25 isolates of the study that did not demonstrate the use of the AdeABC efflux pump as an active mechanism of resistance, while the required set of genes was present, and their mRNA produced.

**Figure 4:**
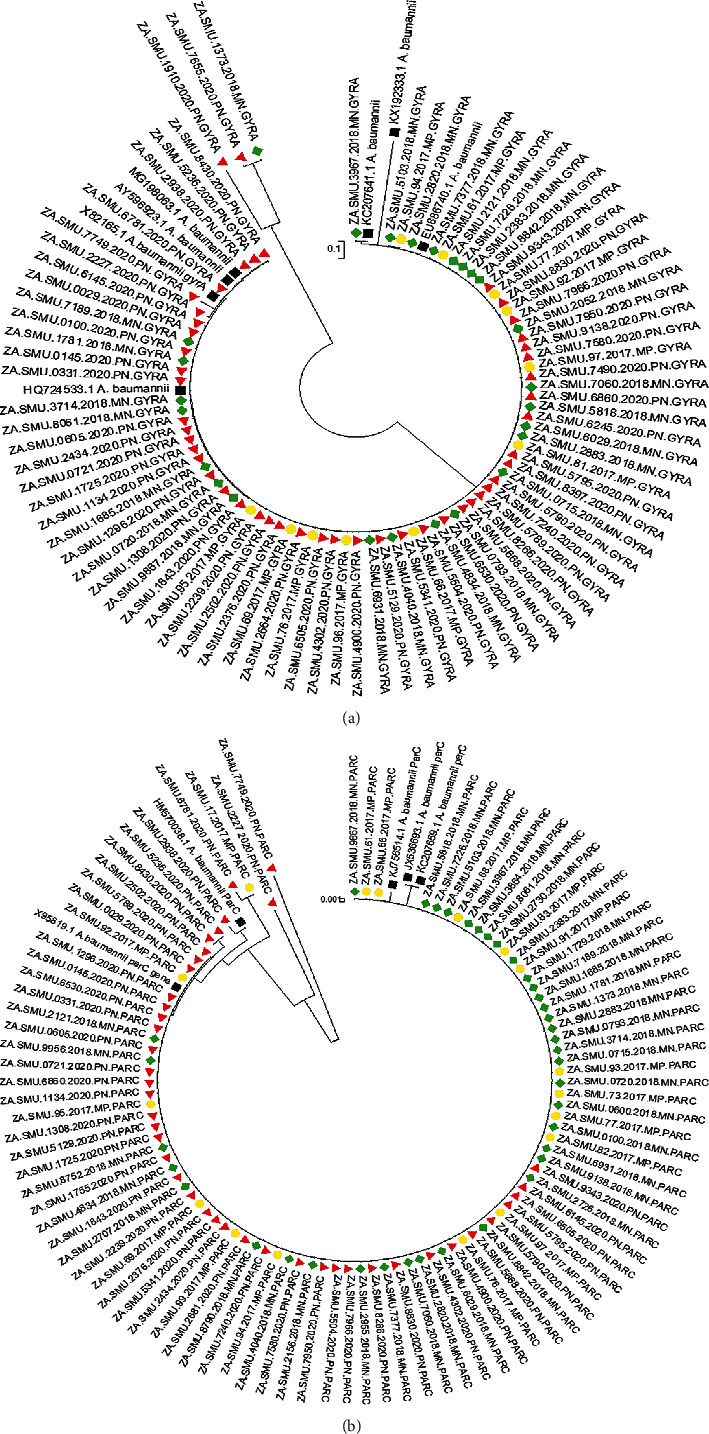
(a) Phylogenetic analysis of the gyrA gene sequences showing highly related strains. The evolutionary history was inferred using the neighbour-joining method [48]. (b) Phylogenetic analysis of the parC gene sequences showing highly related strains. The evolutionary history was inferred using the neighbour-joining method [48].

Quinolone resistance-determining regions are specific regions on the *gyrA* and *parC* genes that code for the amino acid mutations that give *A*. *baumannii* its ability to resist quinolones [38, 39]. These quinolone resistance-determining regions are found on codon 80 (serine 80) and 84 (Glu-84) of the *parC* gene and codon 83 (serine 83) of the *gyrA* gene [40]. In this study, all the clinical isolates (100%) were PCR positive for *gyrA* and 99% positive for *parC* (Table 2; Figure 3; Supplementary 4). The sequencing analysis of *gyrA* revealed that 89% of isolates showed a point mutation in serine 83, 39.2% had mutation on serine 80, and none of isolates exhibits mutation on Glu-84 for *parC*-amplified gene. Several studies reported similar findings suggesting that amino acid substitutions on *gyrA* and *parC* genes are associated with quinolone resistance in *A*. *baumannii* [67, 68]. The phenotypic susceptibility testing of isolated strains of *A*. *baumannii* to ciprofloxacin (83% resistance) correlates with fluoroquinolone resistance-associated genes sequence analysis investigated in this study. However, it is known that other amino acid substitutions in *gyrA* induce *A*. *baumannii* resistance to quinolone. A study reported quinolone resistance mutations at serine 81 on the *gyrA* gene inducing change from serine to leucine [31]. Isolates showing mutations from glycine 81 to valine and alanine 84 to proline on the *gyrA* gene have also been reported to contribute to resistance to quinolone in *A*. *baumannii* [69, 70]. Of all the study isolates, 37 had mutations on both genes, while 10 did not have any mutations (Table 2; Figure 4; Supplementary 4). Presence of mutation in the both *gyrA* and *parC* gene contributes to a higher level of ciprofloxacin resistance rather than a single mutation in the *gyrA* or *parC* gene as reported by Lee and his colleagues [72]. The results of this current study support the report by Lee et al. [72]. The 37 isolates with mutation on both genes revealed higher level resistance to tigecycline than the 10 isolates that did not have any (Table 2; Supplementary 4). These results suggest that single or double mutation on *parC* and/or *gyrA* and their concomitant presence or absence on both genes affect the level of resistance to antibiotics.

The phylogenetic relatedness of the isolates was investigated using the neighbour-joining method [48] on MEGA 4.0 (Figures 4(a) and 4(b)). The analysis showed very minimal evolutionary distance between the isolates from DGMAH. This observation is most likely because of the nature of the genes; *parC* and *gyrA* are housekeeping genes very essential for replication and thus highly conserved.

This study has also observed that mutations within the *A*. *baumannii* isolates have gradually increased over time from 2017 to 2020, implying that isolates at DGMAH are increasingly becoming resistant to drugs used to treat them. Other studies have shown a gradual increase in resistance in healthcare institutions [57, 73, 74].

## 5. Conclusion

Isolates of *A*. *baumannii* at DGMAH demonstrated a high resistance prevalence to available antibiotics. The use of the AdeABC efflux pump system as an active mechanism of resistance combined with point mutation mainly in *gyrA* contributes to broaden the resistance spectrum of *A*. *baumannii* isolates at DGMAH. This situation is particularly alarming as the locally isolated strains demonstrated an increase in resistance to tigecycline. Judicial use of antimicrobials supported by antibiotic susceptibility results should be instituted to control the rise of resistance in *A*. *baumannii* strains.

## Figures and Tables

**Table 1:** Combined analysis of molecular and phenotypic investigations of the active efflux pump.

Efflux pump pattern	Number of samples	Efflux pump active	Efflux pump not active
PCR B^+^R^+^S^+^	101	75	26
qPCR B^+^R^+^S^+^	100	75	25
PCR-qPCR B^+^R^+^S^+^	100	75	25

PCR, polymerase chain reaction; qPCR, quantitative polymerase chain reaction; +, positive PCR.

**Table 2:** Summary of mutation analysis in *parC* and *gyrA* genes.

Mutation in sequence	*gyrA* +	*gyrA* −	Total
*parC* +	37	1	38
*parC* −	53	10	63
Total	90	11	101

There was no mutation in amplified *parC* sequence at serine 84; the data show only mutation at serine 80 for *parC* gene. +, presence of mutation inducing change in amino acid. −, absence of mutation inducing change in amino acid.

## Data Availability

The data used to support the findings of this study are included within the Supplementary Materials.

## Supplementary Materials

Supplementary Materials Supplementary 1. Oligonucleotides sequences for PCR. Supplementary 2. Thermocycling conditions and positive control strains used for polymerase chain reaction amplification of targeted genes. Supplementary 3. Molecular and phenotypic investigations of the active efflux pump as the mechanism of resistance. Supplementary 4. Molecular investigation of mutation in *parC* and *gyrA* as the quinolone resistance mechanism.
