# Supplementary material for: Efflux Pump Activity and Mutations Driving Multidrug Resistance in Acinetobacter baumannii at a Tertiary Hospital in Pretoria, South Africa
Source: Int J Microbiol. 2021 Oct 7;2021:9923816. doi: 10.1155/2021/9923816 (PMC8516574; doi:10.1155/2021/9923816)
Supplement: Supplementary Materials — Supplementary 1. Oligonucleotides sequences for PCR. Supplementary 2. Thermocycling conditions and positive control strains used for polymerase chain reaction amplification of targeted genes. Supplementary 3. Molecular and phenotypic investigations of the active efflux pump as the mechanism of resistance. Supplementary 4. Molecular investigation of mutation in parC and gyrA as the quinolone resistance mechanism. [file 9923816.f1.docx]

SUPPLEMENT MATERIAL

| **Supplement 1** Oligonucleotides sequences for PCR | | | |
| --- | --- | --- | --- |
| **Primers** | **Sequences** | **Genes** | **Sizes (References)** |
| Oxacillinase *bla_OXA-51_* | | | |
| OXA-51 for | TAATGCTTTGATCGGCCTTG | *bla_OXA-51_* | 353bp (75) |
| OXA-51 rev | TGGATTGCACTTCATCTTGG |  |  |
| AdeABC efflux pump system | | | |
| AdeB for | TTAACGATAGCGTTGTAACC | *AdeB* | 541bp (76) |
| AdeB rev | TGAGCAGACAATGGAATAGT |  |  |
| AdeR for | ACTACGATATTGGCGACATT | *AdeR* | 447bp (76) |
| AdeR rev | GCGTCAGATTAAGCAAGATT |  |  |
| AdeS for | TTGGTTAGCCACTGTTATCT | *AdeS* | 544bp (76) |
| AdeS rev | AGTGGACGTTAGGTCAAGTT |  |  |
| Quinolone resistance associated genes (*parC* and *gyrA*) | | | |
| GyrA for | AAATCTGCTCGTGTCGTTGG | *gyrA* | 249bp (77) |
| GyrA rev | GCCATACCTACAGCAATACC |  |  |
| ParC for | AAGCCCGTACAGCGCCGTATT | *parC* | 227bp (77) |
| ParC rev | AAAGTTATCTTGCCATTCGCT |  |  |
| *All oligonucleotides were synthesised and purified by Inqaba Biotechnical Industries, South Africa | | | |
|  |  |  |  |
| References |  |  |  |
| 75. Kock MM, Bellomo AN, Storm N, Ehlers MM. Prevalence of carbapenem resistance genes in Acinetobacter baumannii isolated from clinical specimens obtained from an academic hospital in South Africa. Southern African Journal of Epidemiology and Infection [Internet]. 2013 Jan 1 [cited 2021 Feb 17];28(1):28–32. Available from: https://doi.org/10.1080/10158782.2013.11441516 | | | |
| 76. Beheshti M, Talebi M, Ardebili A, Bahador A, Lari AR. Detection of AdeABC efflux pump genes in tetracycline-resistant Acinetobacter baumannii isolates from burn and ventilator-associated pneumonia patients. J Pharm Bioallied Sci. 2014 Oct;6(4):229–32. | | | |
| 77. Ardebili A, Lari AR, Beheshti M, Lari ER. Association between mutations in gyrA and parC genes of Acinetobacter baumannii clinical isolates and ciprofloxacin resistance. Iran J Basic Med Sci [Internet]. 2015 Jun [cited 2021 Feb 17];18(6):623–6. Available from: https://www.ncbi.nlm.nih.gov/pmc/articles/PMC4509960/ | | | |

**Supplement 2 Thermocycling conditions and positive control strains used for polymerase chain reaction amplification of targeted genes**

| **Supplement 3 Molecular and phenotypic investigation of active efflux pump as mechanism of resistance** | | | | | | | | |
| --- | --- | --- | --- | --- | --- | --- | --- | --- |
| **sample identification** | **PCR** | | | **qRT-PCR** | | | **Phenotypic evaluation of efflux pump** | |
|  |  |  |  |  |  |  | **MIC (µg/mL)** | |
|  | ***adeB*** | ***adeR*** | ***adeS*** | ***adeB*** | ***adeR*** | ***adeS*** | **CCCP -** | **CCCP +** |
| **za.smu.17.2017.mp** | **+** | **+** | **+** | **+** | **+** | **+** | **0,37** | **0,34** |
| **za.smu.29.2020.pn** | **+** | **+** | **+** | **+** | **+** | **+** | **0,25** | **0,19** |
| **za.smu.60.2017.mp** | **+** | **+** | **+** | **+** | **+** | **+** | **0,44** | **0,31** |
| **za.smu.61.2017.mp** | **+** | **+** | **+** | **+** | **+** | **+** | **0,22** | **0,15** |
| **za.smu.66.2017.mp** | **+** | **+** | **+** | **+** | **+** | **+** | **0,38** | **0,25** |
| **za.smu.68.2017.mp** | **+** | **+** | **+** | **+** | **+** | **+** | **0,62** | **0,5** |
| **za.smu.69.2017.mp** | **+** | **+** | **+** | **+** | **+** | **+** | **0,37** | **0,37** |
| **za.smu.70.2017.mp** | **+** | **+** | **+** | **+** | **+** | **+** | **0,019** | **0,016** |
| **za.smu.73.2017.mp** | **+** | **+** | **+** | **+** | **+** | **+** | **0,25** | **0,19** |
| **za.smu.76.2017.mp** | **+** | **+** | **+** | **+** | **+** | **+** | **0,5** | **0,28** |
| **za.smu.77.2017.mp** | **+** | **+** | **+** | **+** | **+** | **+** | **0,19** | **0,25** |
| **za.smu.81.2017.mp** | **+** | **+** | **+** | **+** | **+** | **+** | **0,31** | **0,31** |
| **za.smu.82.2017.mp** | **+** | **+** | **+** | **+** | **+** | **+** | **0,22** | **0,19** |
| **za.smu.83.2017.mp** | **+** | **+** | **+** | **+** | **+** | **+** | **0,38** | **0,25** |
| **za.smu.91.2017.mp** | **+** | **+** | **+** | **+** | **+** | **+** | **0,22** | **0,19** |
| **za.smu.92.2017.mp** | **+** | **+** | **+** | **+** | **+** | **+** | **0,62** | **0,62** |
| **za.smu.93.2017.mp** | **+** | **+** | **+** | **+** | **+** | **+** | **0,22** | **0,15** |
| **za.smu.94.2017.mp** | **+** | **+** | **+** | **+** | **+** | **+** | **0,38** | **0,38** |
| **za.smu.95.2017.mp** | **+** | **+** | **+** | **+** | **+** | **+** | **0,28** | **0,18** |
| **za.smu.96.2017.mp** | **+** | **+** | **+** | **+** | **+** | **+** | **0,37** | **0,37** |
| **za.smu.97.2017.mp** | **+** | **+** | **+** | **+** | **+** | **+** | **0,22** | **0,15** |
| **za.smu.98.2017.mp** | **+** | **+** | **+** | **+** | **+** | **+** | **0,25** | **0,25** |
| **za.smu.99.2017.mp** | **+** | **+** | **+** | **+** | **+** | **+** | **0,22** | **0,22** |
| **za.smu.100.2018.mn** | **+** | **+** | **+** | **+** | **+** | **+** | **0,28** | **0,25** |
| **za.smu.145.2020.pn** | **+** | **+** | **+** | **+** | **+** | **+** | **0,19** | **0,19** |
| **za.smu.331.2020.pn** | **+** | **+** | **+** | **+** | **+** | **+** | **0,44** | **0,19** |
| **za.smu.600.2018.mn** | **+** | **+** | **+** | **+** | **+** | **+** | **0,44** | **0,31** |
| **za.smu.605.2020.pn** | **+** | **+** | **+** | **+** | **+** | **+** | **0,62** | **0,38** |
| **za.smu.715.2018.mn** | **+** | **+** | **+** | **+** | **+** | **+** | **0,28** | **0,15** |
| **za.smu.720.2018.mn** | **+** | **+** | **+** | **+** | **+** | **+** | **0,37** | **0,28** |
| **za.smu.721.2020.pn** | **+** | **+** | **+** | **+** | **+** | **+** | **0,19** | **0,125** |
| **za.smu.793.2018.mn** | **+** | **+** | **+** | **+** | **+** | **+** | **0,42** | **0,42** |
| **za.smu.1134.2020.pn** | **+** | **+** | **+** | **+** | **+** | **+** | **0,5** | **0,38** |
| **za.smu.1296.2020.pn** | **+** | **+** | **+** | **+** | **+** | **+** | **0,19** | **0,19** |
| **za.smu.1308.2020.pn** | **+** | **+** | **+** | **+** | **+** | **+** | **0,22** | **0,19** |
| **za.smu.1373.2018.mn** | **+** | **+** | **+** | **+** | **+** | **+** | **0,34** | **0,17** |
| **za.smu.1685.2018.mn** | **+** | **+** | **+** | **+** | **+** | **+** | **0,38** | **0,25** |
| **za.smu.1725.2020.pn** | **+** | **+** | **+** | **+** | **+** | **+** | **0,19** | **0,19** |
| **za.smu.1729.2018.mn** | **+** | **+** | **+** | **+** | **+** | **+** | **0,44** | **0,44** |
| **za.smu.1755.2020.pn** | **+** | **+** | **+** | **+** | **+** | **+** | **0,38** | **0,25** |
| **za.smu.1781.2018.mn** | **+** | **+** | **+** | **+** | **+** | **-** | **0,34** | **0,37** |
| **za.smu.1843.2020.pn** | **+** | **+** | **+** | **+** | **+** | **+** | **0,38** | **0,38** |
| **za.smu.2052.2018.mn** | **+** | **+** | **-** | **+** | **+** | **-** | **0,44** | **0,5** |
| **za.smu.2121.2018.mn** | **+** | **+** | **+** | **+** | **+** | **+** | **0,22** | **0,19** |
| **za.smu.2156.2018.mn** | **+** | **+** | **+** | **+** | **+** | **+** | **0,44** | **0,22** |
| **za.smu.2227.2020.pn** | **+** | **+** | **+** | **+** | **+** | **+** | **0,047** | **0,032** |
| **za.smu.2239.2020.pn** | **+** | **+** | **+** | **+** | **+** | **+** | **0,25** | **0,19** |
| **za.smu.2376.2020.pn** | **+** | **+** | **+** | **+** | **+** | **+** | **0,5** | **0,56** |
| **za.smu.2383.2018.mn** | **+** | **+** | **+** | **+** | **+** | **+** | **0,5** | **0,25** |
| **za.smu.2434.2020.pn** | **+** | **+** | **+** | **+** | **+** | **+** | **0,31** | **0,25** |
| **za.smu.2502.2020.pn** | **+** | **+** | **+** | **+** | **+** | **+** | **0,38** | **0,22** |
| **za.smu.2681.2020.pn** | **+** | **+** | **+** | **+** | **+** | **+** | **0,38** | **0,38** |
| **za.smu.2707.2018.mn** | **+** | **+** | **+** | **+** | **+** | **+** | **0,5** | **0,5** |
| **za.smu.2726.2018.mn** | **+** | **+** | **+** | **+** | **+** | **+** | **0,25** | **0,125** |
| **za.smu.2730.2018.mn** | **+** | **+** | **+** | **+** | **+** | **+** | **0,44** | **0,15** |
| **za.smu.2820.2018.mn** | **+** | **+** | **+** | **+** | **+** | **+** | **0,56** | **0,37** |
| **za.smu.2883.2018.mn** | **+** | **+** | **+** | **+** | **+** | **+** | **0,31** | **0,15** |
| **za.smu.2938.2020.pn** | **+** | **+** | **+** | **+** | **+** | **+** | **0,05** | **0,032** |
| **za.smu.2955.2018.mn** | **+** | **+** | **+** | **+** | **+** | **+** | **0,31** | **0,15** |
| **za.smu.3714.2018.mn** | **+** | **+** | **+** | **+** | **+** | **+** | **0,38** | **0,25** |
| **za.smu.3864.2018.mn** | **+** | **+** | **+** | **+** | **+** | **+** | **0,44** | **0,31** |
| **za.smu.3967.2018.mn** | **+** | **+** | **+** | **+** | **+** | **+** | **0,5** | **0,38** |
| **za.smu.4040.2018.mn** | **+** | **+** | **+** | **+** | **+** | **+** | **0,22** | **0,19** |
| **za.smu.4302.2020.pn** | **+** | **+** | **+** | **+** | **+** | **+** | **0,19** | **0,125** |
| **za.smu.4834.2018.mn** | **+** | **+** | **+** | **+** | **+** | **+** | **0,5** | **0,44** |
| **za.smu.4900.2020.pn** | **+** | **+** | **+** | **+** | **+** | **+** | **0,57** | **0,25** |
| **za.smu.5103.2018.mn** | **+** | **+** | **+** | **+** | **+** | **+** | **0,25** | **0,18** |
| **za.smu.5129.2020.pn** | **+** | **+** | **+** | **+** | **+** | **+** | **0,5** | **0,38** |
| **za.smu.5236.2020.pn** | **+** | **+** | **+** | **+** | **+** | **+** | **0,057** | **0,027** |
| **za.smu.5341.2020.pn** | **+** | **+** | **+** | **+** | **+** | **+** | **0,44** | **0,38** |
| **za.smu.5504.2020.pn** | **+** | **+** | **+** | **+** | **+** | **+** | **0,22** | **0,19** |
| **za.smu.5668.2020.pn** | **+** | **+** | **+** | **+** | **+** | **+** | **0,19** | **0,19** |
| **za.smu.5789.2020.pn** | **+** | **+** | **+** | **+** | **+** | **+** | **0,039** | **0,023** |
| **za.smu.5790.2020.pn** | **+** | **+** | **+** | **+** | **+** | **+** | **0,44** | **0,25** |
| **za.smu.5795.2020.pn** | **+** | **+** | **+** | **+** | **+** | **+** | **0,38** | **0,25** |
| **za.smu.5818.2018.mn** | **+** | **+** | **+** | **+** | **+** | **+** | **0,31** | **0,31** |
| **za.smu.6029.2018.mn** | **+** | **+** | **+** | **+** | **+** | **+** | **0,25** | **0,19** |
| **za.smu.6145.2020.pn** | **+** | **+** | **+** | **+** | **+** | **+** | **0,44** | **0,38** |
| **za.smu.6505.2020.pn** | **+** | **+** | **+** | **+** | **+** | **+** | **0,62** | **0,5** |
| **za.smu.6530.2020.pn** | **+** | **+** | **+** | **+** | **+** | **+** | **0,25** | **0,15** |
| **za.smu.6781.2020.pn** | **+** | **+** | **+** | **+** | **+** | **+** | **0,039** | **0,032** |
| **za.smu.6860.2020.pn** | **+** | **+** | **+** | **+** | **+** | **+** | **0,38** | **0,25** |
| **za.smu.6931.2018.mn** | **+** | **+** | **+** | **+** | **+** | **+** | **0,31** | **0,25** |
| **za.smu.7060.2018.mn** | **+** | **+** | **+** | **+** | **+** | **+** | **0,31** | **0,22** |
| **za.smu.7189.2018.mn** | **+** | **+** | **+** | **+** | **+** | **+** | **0,44** | **0,31** |
| **za.smu.7226.2018.mn** | **+** | **+** | **+** | **+** | **+** | **+** | **0,38** | **0,25** |
| **za.smu.7240.2020.pn** | **+** | **+** | **+** | **+** | **+** | **+** | **0,25** | **0,19** |
| **za.smu.7377.2018.mn** | **+** | **+** | **+** | **+** | **+** | **+** | **0,37** | **0,28** |
| **za.smu.7580.2020.pn** | **+** | **+** | **+** | **+** | **+** | **+** | **0,25** | **0,19** |
| **za.smu.7749.2020.pn** | **+** | **+** | **+** | **+** | **+** | **+** | **0,032** | **0,032** |
| **za.smu.7950.2020.pn** | **+** | **+** | **+** | **+** | **+** | **+** | **0,25** | **0,19** |
| **za.smu.7966.2020.pn** | **+** | **+** | **+** | **+** | **+** | **+** | **0,19** | **0,25** |
| **za.smu.8061.2018.mn** | **+** | **+** | **+** | **+** | **+** | **+** | **0,31** | **0,25** |
| **za.smu.8266.2020.pn** | **+** | **+** | **+** | **+** | **+** | **+** | **0,38** | **0,25** |
| **za.smu.8430.2020.pn** | **+** | **-** | **+** | **+** | **-** | **+** | **0,032** | **0,032** |
| **za.smu.8752.2018.mn** | **+** | **+** | **+** | **+** | **+** | **+** | **0,44** | **0,31** |
| **za.smu.8790.2018.mn** | **+** | **+** | **+** | **+** | **+** | **+** | **0,25** | **0,38** |
| **za.smu.8830.2020.pn** | **+** | **+** | **+** | **+** | **+** | **+** | **0,25** | **0,19** |
| **za.smu.8842.2018.mn** | **+** | **+** | **+** | **+** | **+** | **+** | **0,62** | **0,44** |
| **za.smu.9138.2018.mn** | **+** | **+** | **+** | **+** | **+** | **+** | **0,25** | **0,19** |
| **za.smu.9343.2020.pn** | **+** | **+** | **+** | **+** | **+** | **+** | **0,31** | **0,31** |
| **za.smu.9867.2018.mn** | **+** | **+** | **+** | **+** | **+** | **+** | **0,31** | **0,37** |
| **za.smu.9956.2018.mn** | **+** | **+** | **+** | **+** | **+** | **+** | **0,18** | **0,18** |
| **+: Positive; -: Negative** |  |  |  |  |  |  |  |  |

| **Supplement 4 Molecular investigation of mutation in *parC* and *gyrA* as quinolone resistance mechanism** | | | | | |
| --- | --- | --- | --- | --- | --- |
| **Sample Identification** | ***parC*** | | | ***gyrA*** | |
|  |  |  |  |  |  |
|  | **PCR** | **Serine-80:** | **Glu-84:** | **PCR** | **Serine-83: Serine** |
|  |  | **Serine to Leucine** | **Glutamic acid** |  | **to Leucine** |
|  |  |  | **To Valine** |  |  |
| **za.smu.17.2017.mp** | **+** | **-** | **-** | **+** | **L** |
| **za.smu.29.2020.pn** | **+** | **-** | **-** | **+** | **L** |
| **za.smu.60.2017.mp** | **+** | **-** | **-** | **+** | **-** |
| **za.smu.61.2017.mp** | **+** | **-** | **-** | **+** | **L** |
| **za.smu.66.2017.mp** | **+** | **-** | **-** | **+** | **L** |
| **za.smu.68.2017.mp** | **+** | **-** | **-** | **+** | **L** |
| **za.smu.69.2017.mp** | **+** | **-** | **-** | **+** | **L** |
| **za.smu.70.2017.mp** | **-** | **-** | **-** | **+** | **-** |
| **za.smu.73.2017.mp** | **+** | **-** | **-** | **+** | **L** |
| **za.smu.76.2017.mp** | **+** | **-** | **-** | **+** | **L** |
| **za.smu.77.2017.mp** | **+** | **-** | **-** | **+** | **L** |
| **za.smu.81.2017.mp** | **+** | **-** | **-** | **+** | **L** |
| **za.smu.82.2017.mp** | **+** | **-** | **-** | **+** | **L** |
| **za.smu.83.2017.mp** | **+** | **-** | **-** | **+** | **L** |
| **za.smu.91.2017.mp** | **+** | **-** | **-** | **+** | **L** |
| **za.smu.92.2017.mp** | **+** | **-** | **-** | **+** | **L** |
| **za.smu.93.2017.mp** | **+** | **-** | **-** | **+** | **L** |
| **za.smu.94.2017.mp** | **+** | **-** | **-** | **+** | **L** |
| **za.smu.95.2017.mp** | **+** | **L** | **-** | **+** | **L** |
| **za.smu.96.2017.mp** | **+** | **-** | **-** | **+** | **L** |
| **za.smu.97.2017.mp** | **+** | **-** | **-** | **+** | **L** |
| **za.smu.98.2017.mp** | **+** | **-** | **-** | **+** | **L** |
| **za.smu.99.2017.mp** | **+** | **-** | **-** | **+** | **-** |
| **za.smu.100.2018.mn** | **+** | **-** | **-** | **+** | **L** |
| **za.smu.145.2020.pn** | **+** | **-** | **-** | **+** | **L** |
| **za.smu.331.2020.pn** | **+** | **L** | **-** | **+** | **L** |
| **za.smu.600.2018.mn** | **+** | **L** | **-** | **+** | **L** |
| **za.smu.605.2020.pn** | **+** | **L** | **-** | **+** | **L** |
| **za.smu.715.2018.mn** | **+** | **-** | **-** | **+** | **L** |
| **za.smu.720.2018.mn** | **+** | **-** | **-** | **+** | **L** |
| **za.smu.721.2020.pn** | **+** | **-** | **-** | **+** | **L** |
| **za.smu.793.2018.mn** | **+** | **L** | **-** | **+** | **L** |
| **za.smu.1134.2020.pn** | **+** | **L** | **-** | **+** | **L** |
| **za.smu.1296.2020.pn** | **+** | **L** | **-** | **+** | **L** |
| **za.smu.1308.2020.pn** | **+** | **L** | **-** | **+** | **L** |
| **za.smu.1373.2018.mn** | **+** | **-** | **-** | **+** | **L** |
| **za.smu.1685.2018.mn** | **+** | **-** | **-** | **+** | **L** |
| **za.smu.1725.2020.pn** | **+** | **L** | **-** | **+** | **L** |
| **za.smu.1729.2018.mn** | **+** | **-** | **-** | **+** | **L** |
| **za.smu.1755.2020.pn** | **+** | **L** | **-** | **+** | ***** |
| **za.smu.1781.2018.mn** | **+** | **-** | **-** | **+** | **L** |
| **za.smu.1843.2020.pn** | **+** | **L** | **-** | **+** | **L** |
| **za.smu.2052.2018.mn** | **+** | **-** | **-** | **+** | **L** |
| **za.smu.2121.2018.mn** | **+** | **-** | **-** | **+** | **L** |
| **za.smu.2156.2018.mn** | **+** | **L** | **-** | **+** | **L** |
| **za.smu.2227.2020.pn** | **+** | **-** | **-** | **+** | **-** |
| **za.smu.2239.2020.pn** | **+** | **-** | **-** | **+** | **L** |
| **za.smu.2376.2020.pn** | **+** | **L** | **-** | **+** | **L** |
| **za.smu.2383.2018.mn** | **+** | **-** | **-** | **+** | **L** |
| **za.smu.2434.2020.pn** | **+** | **L** | **-** | **+** | **L** |
| **za.smu.2502.2020.pn** | **+** | **L** | **-** | **+** | **L** |
| **za.smu.2681.2020.pn** | **+** | **L** | **-** | **+** | ***** |
| **za.smu.2707.2018.mn** | **+** | **-** | **-** | **+** | **-** |
| **za.smu.2726.2018.mn** | **+** | **L** | **-** | **+** | **L** |
| **za.smu.2730.2018.mn** | **+** | **-** | **-** | **+** | **L** |
| **za.smu.2820.2018.mn** | **+** | **L** | **-** | **+** | **L** |
| **za.smu.2883.2018.mn** | **+** | **-** | **-** | **+** | **L** |
| **za.smu.2938.2020.pn** | **+** | **-** | **-** | **+** | **-** |
| **za.smu.2955.2018.mn** | **+** | **-** | **-** | **+** | **L** |
| **za.smu.3714.2018.mn** | **+** | **-** | **-** | **+** | **L** |
| **za.smu.3864.2018.mn** | **+** | **L** | **-** | **+** | **L** |
| **za.smu.3967.2018.mn** | **+** | **L** | **-** | **+** | **L** |
| **za.smu.4040.2018.mn** | **+** | **-** | **-** | **+** | **L** |
| **za.smu.4302.2020.pn** | **+** | **-** | **-** | **+** | **L** |
| **za.smu.4834.2018.mn** | **+** | **-** | **-** | **+** | **L** |
| **za.smu.4900.2020.pn** | **+** | **L** | **-** | **+** | **L** |
| **za.smu.5103.2018.mn** | **+** | **-** | **-** | **+** | **L** |
| **za.smu.5129.2020.pn** | **+** | **-** | **-** | **+** | **L** |
| **za.smu.5236.2020.pn** | **+** | **-** | **-** | **+** | **-** |
| **za.smu.5341.2020.pn** | **+** | **L** | **-** | **+** | **L** |
| **za.smu.5504.2020.pn** | **+** | **-** | **-** | **+** | **L** |
| **za.smu.5668.2020.pn** | **+** | **-** | **-** | **+** | **L** |
| **za.smu.5789.2020.pn** | **+** | **L** | **-** | **+** | **L** |
| **za.smu.5790.2020.pn** | **+** | **L** | **-** | **+** | **L** |
| **za.smu.5795.2020.pn** | **+** | **L** | **-** | **+** | **L** |
| **za.smu.5818.2018.mn** | **+** | **-** | **-** | **+** | **L** |
| **za.smu.6029.2018.mn** | **+** | **-** | **-** | **+** | **L** |
| **za.smu.6145.2020.pn** | **+** | **L** | **-** | **+** | **L** |
| **za.smu.6505.2020.pn** | **+** | **L** | **-** | **+** | **L** |
| **za.smu.6530.2020.pn** | **+** | **L** | **-** | **+** | **L** |
| **za.smu.6781.2020.pn** | **+** | **-** | **-** | **+** | **-** |
| **za.smu.6860.2020.pn** | **+** | **-** | **-** | **+** | **L** |
| **za.smu.6931.2018.mn** | **+** | **-** | **-** | **+** | **L** |
| **za.smu.7060.2018.mn** | **+** | **-** | **-** | **+** | **L** |
| **za.smu.7189.2018.mn** | **+** | **L** | **-** | **+** | **L** |
| **za.smu.7226.2018.mn** | **+** | **L** | **-** | **+** | **L** |
| **za.smu.7240.2020.pn** | **+** | **L** | **-** | **+** | **L** |
| **za.smu.7377.2018.mn** | **+** | **-** | **-** | **+** | **L** |
| **za.smu.7580.2020.pn** | **+** | **L** | **-** | **+** | **L** |
| **za.smu.7749.2020.pn** | **+** | **-** | **-** | **+** | **-** |
| **za.smu.7950.2020.pn** | **+** | **-** | **-** | **+** | **L** |
| **za.smu.7966.2020.pn** | **+** | **L** | **-** | **+** | **L** |
| **za.smu.8061.2018.mn** | **+** | **-** | **-** | **+** | **L** |
| **za.smu.8266.2020.pn** | **+** | **L** | **-** | **+** | **L** |
| **za.smu.8430.2020.pn** | **+** | **-** | **-** | **+** | **-** |
| **za.smu.8752.2018.mn** | **+** | **L** | **-** | **+** | **L** |
| **za.smu.8790.2018.mn** | **+** | **L** | **-** | **+** | **-** |
| **za.smu.8830.2020.pn** | **+** | **L** | **-** | **+** | **L** |
| **za.smu.8842.2018.mn** | **+** | **L** | **-** | **+** | **L** |
| **za.smu.9138.2018.mn** | **+** | **L** | **-** | **+** | **L** |
| **za.smu.9343.2020.pn** | **+** | **-** | **-** | **+** | **L** |
| **za.smu.9867.2018.mn** | **+** | **L** | **-** | **+** | **L** |
| **za.smu.9956.2018.mn** | **+** | **-** | **-** | **+** | **L** |
| **+: Presence; -: Absence; L: Change from Serine to Leucine** | | | | | |
